# Transcriptomic identification of HBx-associated hub genes in hepatocellular carcinoma

**DOI:** 10.7717/peerj.12697

**Published:** 2021-12-22

**Authors:** Zhengzhong Ni, Jun Lu, Weiyi Huang, Hanif Khan, Xuejun Wu, Danmei Huang, Ganggang Shi, Yongdong Niu, Haihua Huang

**Affiliations:** 1 Department of Pharmacology, Shantou University Medical College, Shantou, Guangdong, China; 2 Department of Hepatobiliary Surgery, First Affiliated Hospital of Shantou University Medical College, Shantou, Guangdong, China; 3 Department of Pathology, Second Affiliated Hospital of Shantou University Medical College, Shantou, Guangdong, China

**Keywords:** HBx, Hepatocellular carcinoma, Transcriptomic, Hub genes

## Abstract

**Background:**

Hepatocellular carcinoma (HCC) is one of the most common malignancies around the world. Among the risk factors involved in liver carcinogenesis, hepatitis B virus (HBV) X protein (HBx) is considered to be a key regulator in hepatocarcinogenesis. Whether HBx promotes or protects against HCC remains controversial, therefore exploring new HBx-associated genes is still important.

**Methods:**

HBx was overexpressed in HepG2, HepG2.2.15 and SMMC-7721 cell lines, primary mouse hepatocytes and livers of C57BL/6N mice. High-throughput RNA sequencing profiling of HepG2 cells with HBx overexpression and related differentially-expressed genes (DEGs), pathway enrichment analysis, protein-protein interaction networks (PPIs), overlapping analysis were conducted. In addition, Gene Expression Omnibus (GEO) and proteomic datasets of HBV-positive HCC datasets were used to verify the expression and prognosis of selected DEGs. Finally, we also evaluated the known oncogenic role of HBx by oncogenic array analysis.

**Results:**

A total of 523 DEGs were obtained from HBx-overexpressing HepG2 cells. Twelve DEGs were identified and validated in cells transiently transfected with HBx and three datasets of HBV-positive HCC transcription profiles. In addition, using the Kaplan-Meier plotter database, the expression levels of the twelve different genes were further analyzed to predict patient outcomes.

**Conclusion:**

Among the 12 identified HBx-associated hub genes, HBV-positive HCC patients expressing *ARG1* and *TAT* showed a good overall survival (OS) and relapse-free survival (RFS). Thus, *ARG1* and *TAT* expression could be potential prognostic markers.

## Introduction

Hepatitis B virus (HBV)-associated hepatocellular carcinoma (HCC) is one of the leading malignancies, with a high rate of cancer deaths worldwide (Villanueva, 2019). The HBV X gene (HBx), the smallest of the four open reading frames (ORFs) in the HBV genome, encodes a protein consisting of 154 amino acids and has been shown to be a key regulator in HBV-associated HCC. HBx has multifunctional activities related to hepatocarcinogenesis, including viral DNA replication and infection, host cell proliferation and interactions with various host proteins. Among these proposed processes, HBx can regulate a variety of signaling pathways, including inhibition of apoptosis, promotion of cell proliferation, enhancement of invasion and metastasis, and inhibition of tumor suppressor genes (Du et al., 2012; Lee et al., 2005; Liu et al., 2012; Liu et al., 2010; Xu et al., 2014). With the rapid development of high-throughput sequencing technologies, the mechanism of hepatocellular carcinogenesis has been extensively studied (Brunner et al., 2019; Gao et al., 2019; Jiang et al., 2013; Zhao et al., 2016). However, the understanding of the role of HBx in HCC remains incomplete and controversial.

HBx does not bind directly to host genomic DNA, but its transcriptional activation is linked to direct interaction with its binding partners and the corresponding signaling modulation (Feitelson et al., 1993; Li et al., 2019; Tan, 2011). HBx has been shown to mediate host-cell signaling by directly or indirectly affecting host and viral gene expression, including transactivation and upregulation of *CTTN, CPAP* and *CEBPA*, as well as dysregulation of *K-RAS, PTPN13, RPS7* and *APOBEC3G* (Chen et al., 2017; Kong et al., 2019; Li et al., 2019; Niu et al., 2021b; Pandey & Kumar, 2015; Yan et al., 2021; Yen et al., 2019). In addition, ATX-HBx transgenic mice do not develop spontaneous HCC (Lee et al., 1990). We have reported that HBx-Farnesoid X receptor (FXR) signaling can inhibit spontaneous hepatocarcinogenesis in an ATX-HBx/FXR KO mouse model (Niu et al., 2017). However, some HBx transgenic mice have been shown to develop spontaneous HCC (Kim et al., 1991; Wang et al., 2004), indicating that the precise mechanism of HBx-induced hepatocellular carcinogenesis requires further study.

Accordingly, in the present study, we aimed to identify new HBx-related hub genes in hepatocellular carcinogenesis to fully characterize the expression profile of HBx by determining the transcriptome profile of HBx-overexpressing HCC cell lines by RNA-Seq. In order to further explore the possible molecular mechanisms of HBx-induced HCC, we also provide further insights into HCC progression related to HBx by using the data mining in our data and several public datasets.

## Materials & Methods

### Cell culture

HepG2 cells, HepG2.2.15 cells and SMMC-7721 cells were preserved in our laboratory and HepG2 cells were confirmed by short tandem repeat (STR) analysis. All cell lines were cultured in Dulbecco’s modified Eagle’s medium (DMEM; Gibco, 12800017) with 10% fetal bovine serum (FBS; Gibco, 10099141C) at 37 °C in a 5% CO_2_ incubator. HepG2.2.15 cells were cultured in DMEM growth medium supplemented with G418 (200 µg/mL).

### Cell transfection

Full-length HBx (AF100309.1) was inserted into pcDNA3.0 vector to construct HBx plasmids encoding Flag-tagged proteins. The expression plasmid for Flag-tagged HBx has been previously described (Niu et al., 2013). For electrotransfection, cells were resuspended in PBS buffer at 1.5 × 10^6^ cells per 100 µL, then 3.0 µg plasmid DNA was added. The DNA-cell mixture was transferred to Nucleocuvette™ Vessels and cells were electrotransfected with a Lonza 4D-Nucleofector X-unit system. Preheated medium was added, and the cells were evenly distributed into a 6 cm dish. At 8 h after electrotransfection, medium was replaced with fresh growth medium. Samples were collected within 24 h to extract proteins and nucleic acids.

### RNA extraction, library preparation, RNA sequencing and transcriptome analysis

Total RNA was obtained from HBx-overexpressing HepG2 cells with TRIzol^®^ (Invitrogen, Carlsbad, CA). RNA integrity was assessed using the RNA Nano 6000 Assay Kit of the Bioanalyzer 2100 system (Agilent Technologies, CA, USA). RNA samples were prepared by 1.5 µg total RNA per sample. A sequencing library was generated using a NEBNext^®^ Ultra™ RNA Library Prep Kit for Illumina^®^ (NEB, USA) as recommended by the manufacturer, and index codes were added to sequence the attributes for each sample. The index-coded samples were clustered using a HiSeq 4000 PE Cluster Kit (Illumina) on the cBot Cluster Generation System according to the instructions of the manufacturer. After cluster generation, the library was sequenced on an Illumina HiSeq 4000 platform and 150 bp paired-end reads were generated. RNA-Seq was performed by the Shanghai Lifegenes Technology Co., Ltd. Raw data in FastQ format was first processed using internal Perl scripts. In this process, clean data (clean reads) was obtained by deleting reads containing the adapter, reads containing poly-N, and low-quality reads from the raw data. The contents of Q20, Q30 and GC were calculated. All downstream analyses were based on high quality clean data. The reference genome and gene model notes files can be downloaded directly from the Genome website. HISAT2 V2.1.0 was used to compare the paired end clean reads with the reference genome. HTSeq V0.11.2 was used to calculate the number of reads for each gene. The FPKM of each gene was then calculated based on the gene length and the reads count plotted on the gene. A differentially-expressed genes (DEGs) list was created based on the DEGseq R package (1.28.0); *P* < 0.05 and Fold Change (FC) > 1.5 were used as the cut-off criteria.

### Microarray data

Four expression profiling datasets GSE121248 (Wang, Ooi & Hui, 2007), GSE55092 (Melis et al., 2014), GSE84402 (Wang et al., 2017) and GSE14520 (Roessler et al., 2010) were downloaded from GEO (Edgar, Domrachev & Lash, 2002). The probe number was converted to the gene symbol based on the annotation information in the platform. The GSE84402 dataset contained 14 HBV-associated HCC tissues and corresponding non-cancerous tissues. GSE121248 contained 70 HBV-associated HCC and 37 adjacent non-cancerous tissues. GSE55092 contained 10 HBV-associated HCC tissues and corresponding non-cancerous tissues. GSE14520 contained 212 HBV-associated HCC tissues with clinical survival information, and MedCalc (version 20.0.3) software was used for receiver operating characteristic (ROC) analysis to determine the role of hub genes in the diagnosis of HBV-associated HCC.

### Identification of DEGs

To identify DEGs, we used GEO2R (https://www.ncbi.nlm.nih.gov/geo/geo2r/) to compare HBV-associated HCC and adjacent nontumor tissue transcription profiles. A list of DEGs was created using the limma R package in GEO2R; *P* <  0.05 and Fold Change >2.0 were used as the cut-off criteria.

### GO and KEGG pathway analysis

GO and KEGG pathway analyses of DEGs were performed using the DAVID (https://david.ncifcrf.gov/) database. *P* < 0.05 was considered statistically significant.

### Construction of a protein-protein interaction network (PPI) network and module analysis

We used the STRING database to construct a PPI network of DEGs (Szklarczyk et al., 2019), and a combined score >0.7 was used as the criterion for statistical significance. The PPI network was mapped with Cytoscape and the MCODE plug-in was used to identify the most important module in the PPI network (Bandettini et al., 2012; Su et al., 2014). Selection criteria were as follows: MCODE scores > 5, degree cut-off = 2, node score cut-off = 0.2, Max depth = 100 and k-score = 2.

### Hydrodynamic gene delivery

All C57BL/6N mice were obtained from Beijing Vital River Laboratory Animal Technology Co., Ltd (Beijing, China). Mice were housed in individual ventilated cages (23 ± 3 °C, 40–70% humidity, 12-hour light/dark cycle) with free access to food and water in a specific pathogen-free (SPF) laboratory animal room. Animals were randomly assigned to an HBx group or empty vector group. Hydrodynamic gene delivery of HBx (*n* = 5) or empty vector (*n* = 5) was performed on 6- to 8-week-old male mice as previously described (Niu et al., 2017). Mice were euthanized by CO_2_ asphyxiation 24 h after the treatment. Livers were excised, quickly frozen with dry ice, and stored at −80 °C. Subsequently, total RNA was extracted from liver tissues and gene expression was detected by qRT-PCR. Mice that successfully overexpressed HBx were selected for data analysis (CT ≤ 20). All experiments were carried out in the Laboratory Animal Center of Shantou University Medical College. All protocols and procedures were approved by the Institutional Animal Care and Research Advisory Committee of Shantou University Medical College (SUMC2015-069 and SUMC2021-212).

### Primary mouse hepatocyte isolation

Mice were anesthetized with 1% pentobarbital sodium (7.5 mL/kg) and disinfected with alcohol. The abdominal cavity of the mice was opened along the lower abdomen in a U-shaped incision. The inferior vena cava was separated, a venous indwelling needle was inserted and fixed, while the superior vena cava was ligated and the portal vein was severed. EGTA 3 min, protease (14 mg/mouse) 5–6 min and collagenase D (3.7 U/mouse) 7 min were sequentially infused at 42 °C for liver digestion. After digestion, the whole liver was removed and placed in the remaining collagenase D, and the digestion was continued at 42 °C for 5 min. The liver was placed on a super-clean platform, part of the collagenase D was discarded and the separated hepatocytes were transferred to a 10 cm dish. The appropriate amount of DMEM medium was added and the liver was minced using forceps. The cell suspension was filtered through a cell filter, collected in a 50 mL centrifuge tube, and centrifuged three times for 3 min each at 50 g (4 °C). Supernatant was gently aspirated, and 20 mL DMEM medium was added for re-suspension. The cell suspension was gently added to the layering solution, centrifuged or 10 min at 400 g (4 °C) without brake and the upper dead cells were removed by aspiration, 15 mL DMEM was added for re-suspension and counting, and 3  × 10^5^ cells were seeded in each well of a 6-well plate. Fresh medium was replaced after 7 h. HBx overexpression was performed. All DMEM medium contained 10% fetal bovine serum.

### RNA extraction and quantitative real-time PCR (qRT-PCR) validation

To verify the DEGs identified in the overlap analysis, we performed qRT-PCR on selected genes. Total RNA was extracted from HepG2 cells using RNAiso Plus Reagent (Invitrogen, Carlsbad, CA). Subsequently, qRT-PCR primers were purchased from BGI (Guangzhou, China). TB Green Premix Ex Taq (TaKaRa, RR820A) was used for 3 repeats of qRT-PCR. Cyclophilin was used as the internal control. The fold change of the RNA level in each sample was measured by the 2^−ΔΔCT^ method and compared to the control sample. Primers are listed in Table 1.

**Table 1:** List of primers used in the quantitative real-time PCR analysis.

Gene	Forward primer	Reverse primer
hALDH8A1	5′-CGACCCATCAACAGGGGAAG-3′	5′-CGACCCATCAACAGGGGAAG-3′
hALDOB	5′-TGTCTGGTGGCATGAGTGAAG-3′	5′-GGCCCGTCCATAAGAGAAACTT-3′
hANGPTL6	5′-GGTTCCGGTCCGTCTTGTG-3′	5′-CCCACTCGCAGTTCATACACT-3′
hARG1	5′-TGGACAGACTAGGAATTGGCA-3′	5′-CCAGTCCGTCAACATCAAAACT-3′
hC8a	5′-AAGGTGAACCAGAGAGTAAGACG-3′	5′-CGGTGTCGGTACTTTTTGTCC-3′
hCPEB3	5′-GAGTCCAGCGTATCCGAAGC-3′	5′-GAGCGGTGATTCCATCTGCAT-3′
hCTSD	5′-CACCACAAGTACAACAGCGAC-3′	5′-CCCGAGCCATAGTGGATGT-3′
hCyclophilin	5′-TGGTGTTTGGCAAAGTGAAA-3′	5′-TCGAGTTGTCCACAGTCAGC-3′
hFAM110C	5′-GGCCGAGTCTGACACCTTC-3′	5′-GGCGTTCCTCTCGATGACC-3′
hGNAL	5′-AGCCCCTATCACTGACTTTGA-3′	5′-CCTTCACGCCTTCATCGTC-3′
hMAGEA6	5′-AGGGGAGGGAAGACAGTATCT-3′	5′-AAAGCCCACTCATGCAGGAG-3′
hPALM2	5′-GACGAAAAAGGTGCTAGGCTAT-3′	5′-CGTCCGTCACTGTCTTCTCC-3′
hRGS5	5′-GACATGGCCCAGAAAAGAATCC-3′	5′-CACAAAGCGAGGCAGAGAATC-3′
hSERPINE1	5′-ACCGCAACGTGGTTTTCTCA-3′	5′-TTGAATCCCATAGCTGCTTGAAT-3′
hTAT	5′-CTGGACTCGGGCAAATATAATGG-3′	5′-GTCCTTAGCTTCTAGGGGTGC-3′
HBx	5′-GACCGACCTTGAGGCATACTT-3′	5′-TGCCTACAGCCTCCTAGTACA-3′
mALDH8A1	5′-CTGCTGGGAATACCGTGATAGC-3′	5′-GTACCTCGGGGTGAGACAC-3′
mALDOB	5′-GAAACCGCCTGCAAAGGATAA-3′	5′-GAGGGTCTCGTGGAAAAGGAT-3′
mANGPTL6	5′-CTGGGCCGTCGTGTAGTAG-3′	5′-CAGTCCTCTAGGAGTATCAGCAG-3′
mARG1	5′-TTGGGTGGATGCTCACACTG-3′	5′-GTACACGATGTCTTTGGCAGA-3′
mC8A	5′-GGGACCCCTGGAGACGAAA-3′	5′-GCCCACAACGACAGGCATTA-3′
mCPEB3	5′-CCAAGCCCGAAGACAGTAGC-3′	5′-CGCGTTTGTAGTGCCTGTG-3′
mCTSD	5′-GCTTCCGGTCTTTGACAACCT-3′	5′-CACCAAGCATTAGTTCTCCTCC-3′
mCyclophilin	5′-GGCTGAGAACGGGAAGCTTGTCAT-3′	5′-CAGCCTTCTCCATGGTGGTGAAGA-3′
mFAM110C	5′-CTGGACTCGCTTGCTAGAATG-3′	5′-GATACAGGACCCCGGCTAGAT-3′
mGNAL	5′-GTCTGGTTGACTACACACCCA-3′	5′-GCCACGTAAATGATCGCAGTG-3′
mMAGEA6	5′-CCCAAGGGCTCTTGCAGAAA-3′	5′-AATGGTCAGAGAAATTGGAGCAT-3′
mPALM2	5′-GAGGCGGAATTGCACAAGGA-3′	5′-GTACCTGTTCGTCAAGCTGTC-3′
mRGS5	5′-CGCACTCATGCCTGGAAAG-3′	5′-TGAAGCTGGCAAATCCATAGC-3′
mSERPINE1	5′-CAAGCTCTTCCAGACTATGGTG-3′	5′-ACCTTTGGTATGCCTTTCCAC-3′
mTAT	5′-TGCTGGATGTTCGCGTCAATA-3′	5′-CGGCTTCACCTTCATGTTGTC-3′

**Notes.**

h, human; m, mouse.

### Western blotting

Cells were lysed using RIPA buffer with 1% phenylmethanesulfonyl fluoride (Beyotime Biotechnology, Jiangsu, China). Protein concentration was measured using the BCA Protein Assay Kit (Invitrogen, Waltham, MA, USA). Protein (20 µg) was separated on 12% SDS-PAGE, and then transferred to PVDF membranes (Merck Millipore, Tullagreen, Carrigtwohill, Co. Cork, Ireland). Membranes were blocked with 5% skim milk for 1 h and washed three times with TBST. The membranes were incubated overnight with primary anti-HBx (#ab39716; 1:1000; Abcam, Cambridge, MA, USA) and anti-*β*-actin antibodies (#BM0627; 1:20000; Boster, Wuhan, China) at 4 °C. Then, membranes were washed and incubated with HRP-conjugated secondary antibody at room temperature for 1 h. Proteins were visualized by ECL (Invitrogen, Waltham, MA, USA).

### Proteomic and immunohistochemical data and oncogenic array

Protein expression of HBx-associated DEGs were viewed using the National Omics Data Encyclopedia (NODE) database OEP000321 (Gao et al., 2019). This dataset included the proteomics information of 159 paired HBV-positive HCC tumors and adjacent non-tumor liver tissues. In short, the obtained differentially-expressed proteins in this dataset were overlapped with 12 hub genes, and six proteins were selected, these six proteins’ OS and RFS analyses were further performed. MedCalc (version 20.0.3) software was used for receiver operating characteristic (ROC) analysis to determine the role of overlapped proteins in the diagnosis of HBV-associated HCC.

Protein expression of HBx-associated DEGs were viewed using the Human Protein Atlas (HPA) database (Thul & Lindskog, 2018). The procedure included inputting gene name, selecting tissue and pathology, and obtaining immunohistochemistry data of normal tissue and cancer tissue. According to the manufacturer’s instructions, proteome profiler array analysis was executed using the Human XL Tumor Array Kit (R&D Systems, Minneapolis, MN).

### Survival data from Kaplan Meier plotter

The Kaplan–Meier plotter (KM plotter) database (https://kmplot.com/analysis/) was used to analyze the prognostic value of overlapped DEGs in HCC (Nagy et al., 2018). In short, the overlapped DEGs were entered into the database and a Kaplan–Meier survival graph was determined. *P* < 0.05 was considered statistically significant.

### Statistical analysis

Experimental data values are expressed as the mean ± standard deviation. Statistical analyses were performed using GraphPad 9. The significance of any difference was determined by independent samples t-tests. *P* < 0.05 was considered statistically significant (∗*P* < 0.05, ∗ ∗*P* < 0.01, ∗ ∗ ∗*P* < 0.001, ∗ ∗ ∗ ∗*P* < 0.01).

## Results

### DEGs were identified in HepG2 cells with HBx overexpression

**Figure 1:**
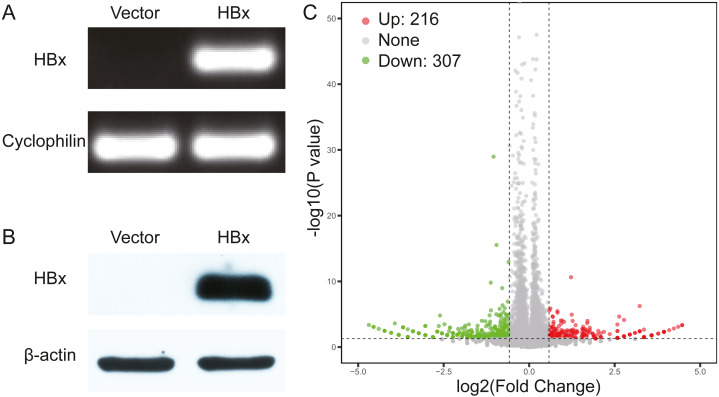
Validation data for HBx-overexpressing HepG2 cell. (A) Agarose gel electrophoresis of RT-PCR amplification products from vector and HBx group. (B) Expression of HBx in vector and HBx group (cell lysates 20 µg) revealed by Western blotting. β-actin was used as an internal control. (C) Volcano plots of DEGs between vector and HBx group.

We initially overexpressed HBx in HepG2 cells and confirmed the overexpression of HBx at both mRNA and protein levels (Figs. 1A, 1B). After analysis of the RNA-Seq data, 523 DEGs were identified in HBx-overexpressing HepG2 cells and included 307 downregulated and 216 upregulated genes compared to HepG2 cells transfected with empty vector (Fig. 1C). The top 10 upregulated DEGs were *MMP1, RABAC1*, *FKBP11, ADM, SLC35E4, PIP4P2, ANAPC10, ZNF358, NRG1* and *SDHAF2*. The top 10 downregulated DEGs were *ZBTB20, POMK, SYNE2, ZNF460, ANKRD36C, KCTD7, CASTOR2, KLHL11, PFKFB1* and *CCL15* (Table 2).

**Table 2:** The top 10 up- and down-regulated genes in HepG2 cells with HBx overexpression.

Gene symbol	logFC	*P* value	Gene symbol	logFC	*P* value
MMP1	1.22	2.35E−11	ZBTB20	−1.04	1.07E−29
RABAC1	3.23	5.63E−07	POMK	−0.95	2.89E−16
FKBP11	0.62	1.31E−06	SYNE2	−0.6	1.12E−13
ADM	0.59	2.56E−06	ZNF460	−1.12	1.54E−10
SLC35E4	0.83	3.4E−06	ANKRD36C	−0.79	1.06E−09
PIP4P2	0.83	5.77E−06	KCTD7	−0.63	1.2E−07
ANAPC10	1.24	1.15E−05	CASTOR2	−0.78	4.39E−07
ZNF358	0.68	2.18E−05	KLHL11	−0.71	1.17E−06
NRG1	2.63	2.27E−05	PFKFB1	−0.86	1.56E−06
SDHAF2	0.69	2.45E−05	CCL15	−1.12	2.59E−06

### GO and KEGG pathway enrichment analysis and construction of a PPI network of the HBx-associated DEGs

To analyze the biological function of screened HBx-associated differentially-expressed genes in HepG2 cells, we used DAVID for function and pathway enrichment analysis. GO enrichment analysis showed that HBx-associated DEGs were enriched in 54 biological processes (BPs), 17 cellular components (CCs) and 39 molecule functions (MFs) (Fig. S1A). Moreover, KEGG pathway enrichment analysis of HBx-associated DEGs were significantly enriched in seven pathways (Fig. S1B). To explore the HBx-associated differential signaling, we used the STRING database to construct a PPI network of DEGs (Fig. S2A). The PPI network included 165 nodes (genes) and 244 edges (interactions). Cytoscape was used to identify the potential important module for HBx-driven carcinogenesis (Fig. S2B). In this module, 5 up-regulated DEGs and 10 down-regulated DEGs were included.

### The overlapping HBx-associated DEGs was observed in cellular and clinical datasets

**Figure 2:**
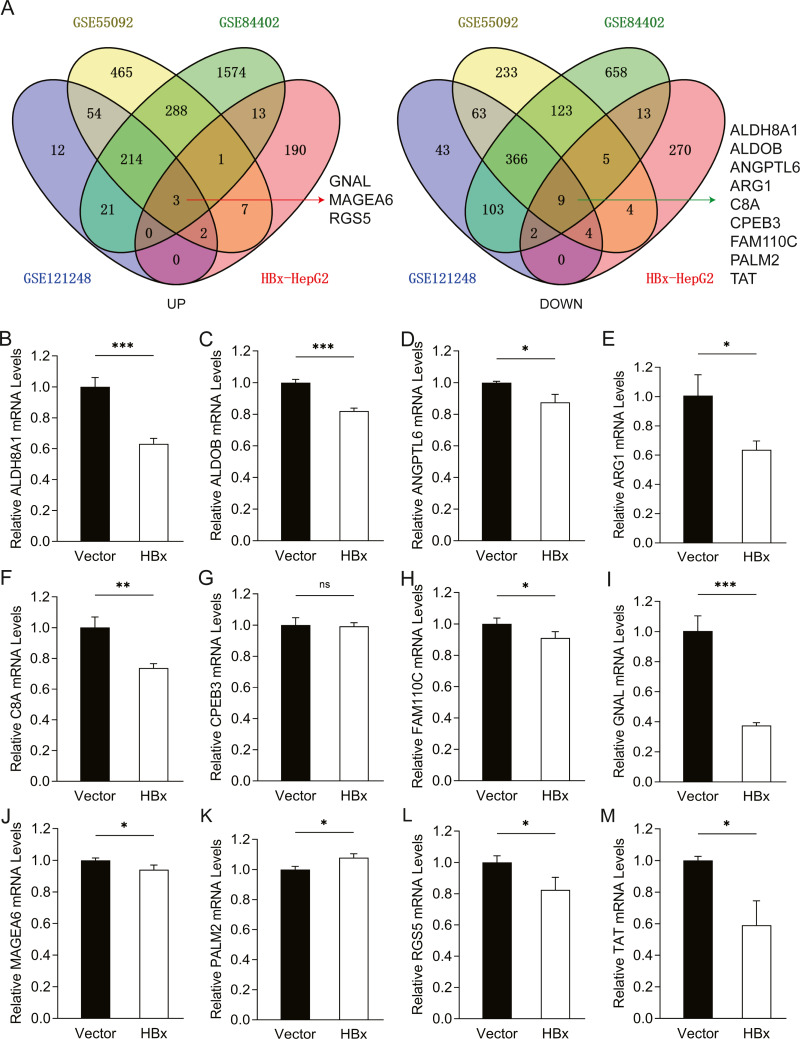
Common genes identification and expression verification. (A) Twelve common DEGs were identified from four datasets. Three commonly up-regulated DEGs and nine commonly down-regulated DEGs. Each color represents a dataset. The crossover region represents the overlapped DEGs. The mRNA expression of (B) *ALDH8A1*, (C) *ALDOB*, (D) *ANGPTL6*, (E) *ARG1*, (F) *C8A*, (G) *CPEB3*, (H) *FAM110C*, (I) *GNAL*, (J) *MAGEA6,* (K) *PALM2,* (L) *RGS5* and (M) *TAT* in vector and HBx group.

**Figure 3:**
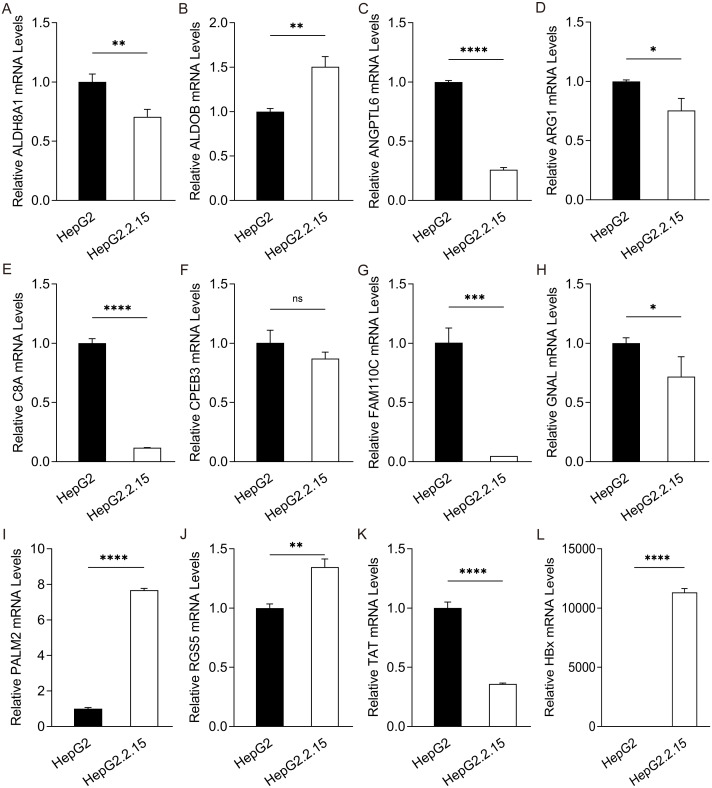
Expression of hub genes in HepG2.2.15 cells. The mRNA expression of (A) *ALDH8A1*, (B) *ALDOB*, (C) *ANGPTL6*, (D) *ARG1*, (E) *C8A*, (F) *CPEB3*, (G) *FAM110C*, (H) *GNAL*, (I) *PALM2,* (J) *RGS5,* (K) *TAT* and (L) *HBx* in HepG2 cells and HepG2. 2.15 cells with stable HBV expression.

To further confirm the candidate HBx-associated hub genes, we also compared their expression levels among the HBV-positive HCC patient clinical data from GSE121248, GSE55092 and GSE84402. After normalization of the microarray data, twelve genes were selected by overlapping analysis, of which three were upregulated DEGs and nine were downregulated DEGs (Fig. 2A). Related information of the 12 candidate DEGs is shown in Table 3. These gene expression changes were subsequently verified in HBx-overexpressing HepG2 cells. The expression levels of *ALDH8A1, ALDOB, ANGPTL6, ARG1, C8A, FAM110C* and *TAT* were consistent with our RNA-Seq data, whereas the trends of *GNAL, MAGEA6, PALM2* and *RGS5* were contrary (Figs. 2B–2M). We also checked the expression levels of 12 hub genes in HepG2.2.15 cells derived from HepG2 cells stably transfected with HBV (Fig. 3). The expression levels of *ALDH8A1, ANGPTL6, ARG1, C8A, FAM110C*, *RGS5* and *TAT* were similar to the results from our RNA-Seq data. In addition, overexpression of HBx in SMMC-7721 cells, livers of C57 mice and primary mouse hepatocytes showed that the expression of *ALDOB, FAM110C* and *TAT* were downregulated in HBx-overexpressing SMMC-7721 cells (Fig. S3). *Tat* and *C8a* were upregulated in hydrodynamic gene delivery mouse models (Fig. S4). *Aldob, Arg1* and *Palm2* were downregulated in fresh separated primary mouse hepatocytes (Fig. S5).

**Table 3:** The basic information of twelve overlapping genes.

Gene symbol	Full name	Tissue enriched
ALDH8A1	Aldehyde dehydrogenase 8 family member A1	kidney, liver
ALDOB	Aldolase, fructose-bisphosphate B	intestine, kidney, liver
ANGPTL6	Angiopoietin like 6	liver
ARG1	Arginase 1	liver
C8A	Complement C8 alpha chain	liver
CPEB3	Cytoplasmic polyadenylation element binding protein 3	brain, liver
FAM110C	Family with sequence similarity 110 member C	ductus deferens
GNAL	G protein subunit alpha L	brain
MAGEA6	MAGE family member A6	testis
PALM2	Paralemmin 2	brain, liver
RGS5	Regulator of G protein signaling 5	Low tissue specificity
TAT	Tyrosine aminotransferase	liver

Subsequently, the protein expression of ALDH8A1, ALDOB, ARG1, FAM110C and GNAL were confirmed by the cancer and normal tissue microarrays (TMAs) data from the Human Protein Atlas (https://www.proteinatlas.org). Here, reduced expression of ALDH8A1, ALDOB and ARG1 were also shown in tumor tissues. However, differences in the expressions of FAM110C and GNAL were not significant (Fig. S6A). Simultaneously, we also compared the oncogenic array between HBx and control in order to further confirm the novel hub genes. We also showed that cathepsin D and serpin E1 were increased by HBx (Fig. S6B).

### Effect of HBx-associated DEGs on HCC patient survival

**Figure 4:**
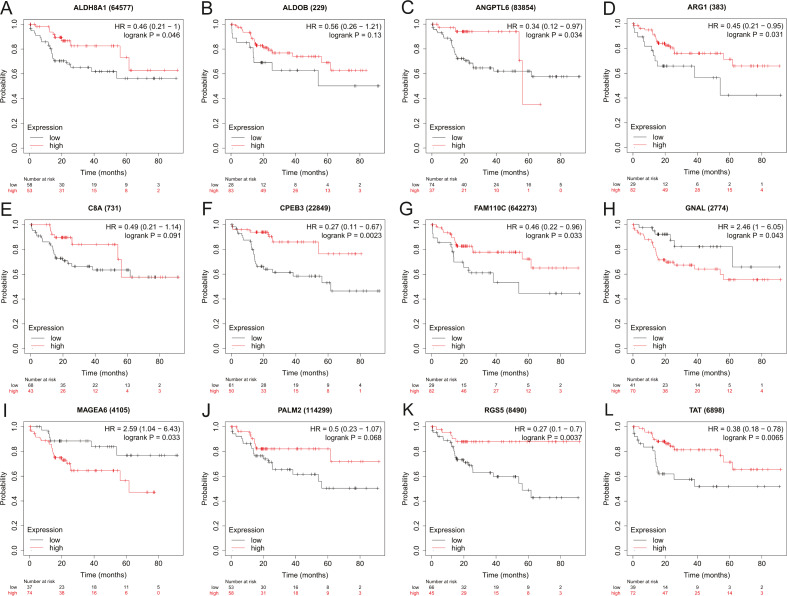
The overall survival analysis of 12 hub genes in HBV-associated HCC patients. The overall survival of (A) *ALDH8A1*, (B) *ALDOB*, (C) *ANGPTL6*, (D) *ARG1*, (E) *C8A*, (F) *CPEB3*, (G) *FAM110C*, (H) *GNAL*, (I) *MAGEA6*, (J) *PALM2*, (K) *RGS5* and (L) *TAT* are presented.

**Figure 5:**
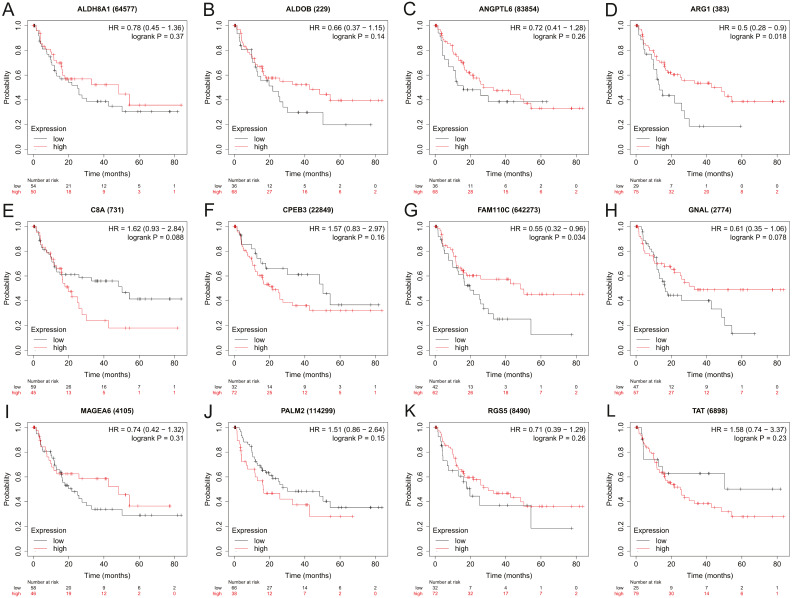
The relapse-free survival analysis of 12 hub genes in HBV-associated HCC patients. The relapse-free survival of (A) *ALDH8A1*, (B) *ALDOB*, (C) *ANGPTL6*, (D) *ARG1*, (E) *C8A*, (F) *CPEB3*, (G) *FAM110C*, (H) *GNAL*, (I) *MAGEA6,* (J) *PALM2*, (K) *RGS5* and (L) *TAT* are presented.

**Figure 6:**
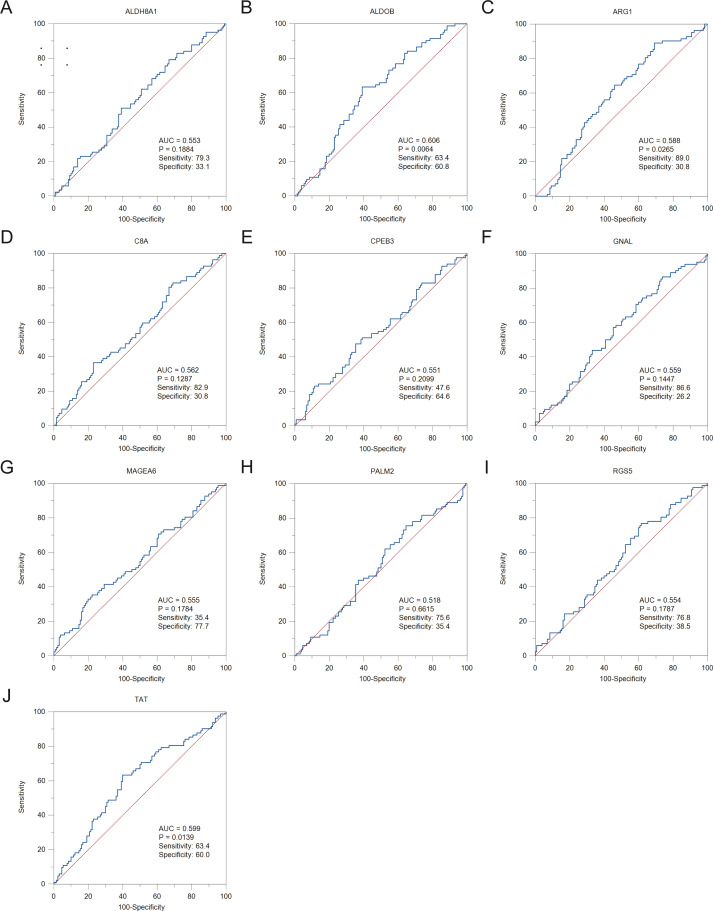
The ROC curves and AUC were used to evaluate the efficiency of hub genes in the diagnosis of HBV-associated HCC. The ROC curves of (A) *ALDH8A1*, (B) *ALDOB*, (C) *ARG1*, (D) *C8A*, (E) *CPEB3*, (F) *GNAL*, (G) *MAGEA6*, (H) *PALM2*, (I) *RGS5* and (J) *TAT* are presented. ROC: receiver operating characteristic; AUC: area under the curve.

To assess the potential prognostic value of the twelve new candidate genes in HBV-associated HCC, the KM plotter was utilized. Low expression levels of *ALDH8A1, ANGPTL6, ARG1, CPEB3, FAM110C, RGS5* and *TAT* were negatively associated with overall survival (OS) in HBV-associated HCC patients, and we observed that low expression levels of *GNAL* and *MAGEA6* were positively associated with OS (Fig. 4). High expression of *ARG1* and *FAM110C* were positively associated with relapse-free survival (RFS) (Fig. 5). To further explore the diagnostic value of these 12 hub genes, we constructed the ROC curve by using the GSE14520 dataset. Three hub genes (*ALDOB, ARG1* and *TAT*) had potential predictive value (*P* < 0.05, AUC > 0.5) (Fig. 6). Since the expressions of *ANPTL6* and *FAM110C* were not listed in the GSE14520 dataset, ROC analysis could not be performed.

### Proteomic analysis of HBx-associated DEGs

**Figure 7:**
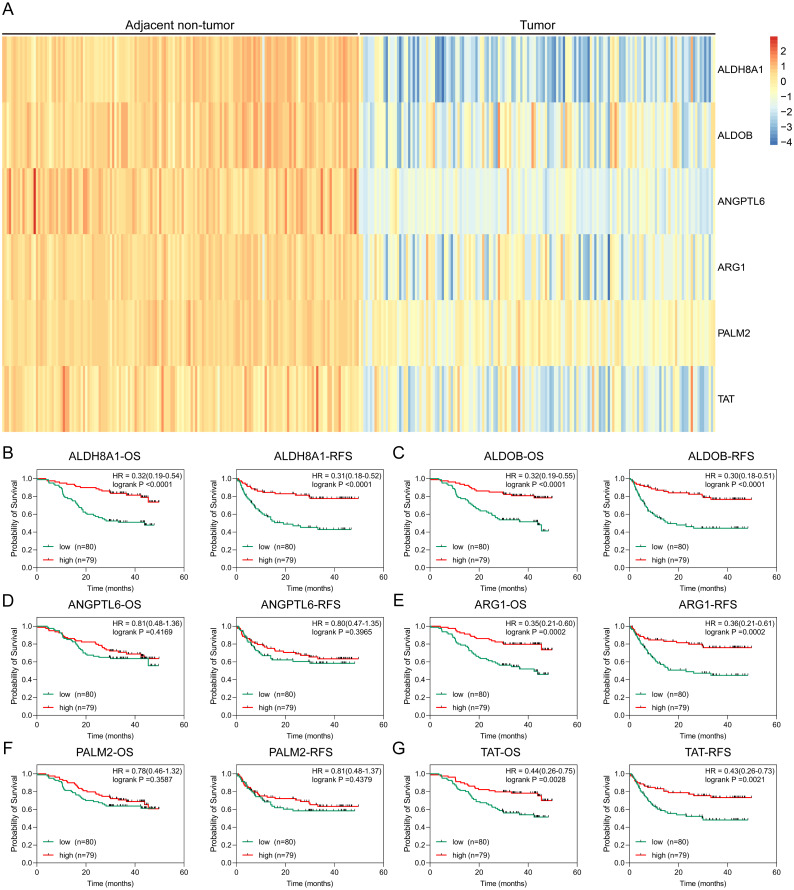
Proteomic analysis of HBx-associated DEGs. (A) The heatmap of six differentially-expressed proteins of HBV-positive HCC. The OS and RFS of (B) ALDH8A1, (C) ALDOB, (D) ANGPTL6, (E) ARG1, (F) PALM2A and (G) TAT are presented.

**Figure 8:**
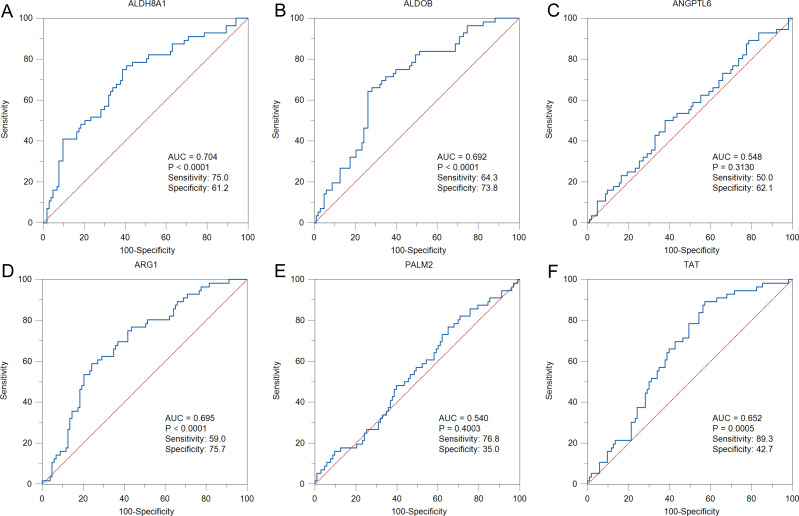
The ROC curves and AUC were used to evaluate the efficiency of six differentially-expressed proteins in the diagnosis of HBV-associated HCC. The ROC curves of (A) ALDH8A1, (B) ALDOB, (C) ANGPTL6, (D) ARG1, (E) PALM2 and (F) TAT are presented.

To further solid the possibility of these hub genes as the potential biomarker of HBV-positive HCC, we compared the protein expression level of 12 candidate DEGs from of HBV-positive HCC patients on public National Omics Data Encyclopedia (NODE) database (OEP000321), and showed ALDH8A1, ALDOB, ARG1, ANGPTL6, PALM2 and TAT were significantly downregulated in HBV-positive HCC tumor tissues compare with corresponding adjacent non-tumor tissues (Fig. 7A). In addition, survival analysis also showed high protein expression levels of ALDH8A1, ALDOB, ARG1 and TAT were positively correlated with OS and RFS (Figs. 7B–7G). A ROC curve was constructed to further explore the diagnostic value of these 6 proteins. Four of the proteins (ALDH8A1, ALDOB, ARG1 and TAT) had potential predictive value (*P* < 0.05, AUC > 0.5) (Fig. 8).

## Discussion

HBV-associated HCC is one of the major malignancies with high mortality worldwide. HBx is an important etiological factor of HBV-associated HCC. Most available evidence suggests a correlation between the presence of HBx and the development of HCC (Niu et al., 2021a). HBV, whether alone or in the wake of other risk factors, is responsible for increased risk of HCC. On average, the relative risk of HBV is at least fivefold (El-Serag & Kanwal, 2014). HBx is not only detected with high frequency in clinical HCC samples, HBx has been demonstrated to have transforming and self-renewal capacity, and to enhance growth factor related signaling, cell differentiation, and angiogenesis in the development and progression of HCC (Chaturvedi et al., 2019; Hwang et al., 2003; Zhang et al., 2009). HBx transgenic mice spontaneously develop tumors (Kim et al., 1991; Wang et al., 2004). However, we have also shown that HBx-FXR signaling can reduce spontaneous hepatocarcinogenesis in a mouse model. Due to the poor understanding of the pathogenesis of HCC induced by HBx, it is still necessary to find novel HBx associated genes or proteins in HBV-positive hepatocellular carcinogenesis.

In our study, a total of 523 DEGs including 307 downregulated and 216 upregulated genes were identified in HBx-overexpressing HepG2 cells. There were 117 signaling pathways identified by enrichment analysis of GO and KEGG between DEGs (FC > 1.5). The pathway analysis showed that HBx-associated enriched pathways are mainly enriched in the Notch signaling pathway, integral components of plasma membrane, positive regulation of GTPase activity and calcium ion binding.

We analyzed the overlapped genes among HBx-overexpressing cells and three clinical datasets of HBV-associated HCC. A total of 12 hub genes were identified. Among the 12 HBx-associated hub genes, *ARG1*, *C8A* and *FAM110C* have previously been reported to be associated with HBV-positive HCC. Recently, high expressed *ANGPTL6* (Hu et al., 2021) is found to be associated with poor prognosis in HCC. The expression levels of *ALDH8A1, ALDOB, CPEB3, GNAL*, *MAGEA6*, *RGS5* and *TAT* in clinical samples shared the same trends in our RNA-Seq data. However, we failed to find some evidence to show the associations between *PALM2* with HBV-positive HCC in previous work. In our subsequent validation, the expression of 12 genes in the three human liver cancer cell lines and primary mouse hepatocytes was not completely consistent with HBx overexpression. For instance, *FAM110C* and *TAT* were downregulated after HBx overexpression in human liver cancer cell lines and consistent with our RNA-Seq data, but there was no significant change found in primary mouse hepatocytes following HBx overexpression. This result may be due to the differences in gene expression between various cell lines. However, in the HBx hydrodynamic gene delivery model, most of the 12 genes showed no significant changes, which can be caused by individual differences in mice.

Aldehyde dehydrogenase eight family member A1 (*ALDH8A1*) is located on human chromosome 6q23.3 and encodes a protein of 53 kDa. It has been implicated in the synthesis of 9-cis-retinoic acid and in the breakdown of the amino acid tryptophan (Davis et al., 2018). *ALDH8A1* is found decreased in nonalcoholic steatohepatitis (NASH), cirrhosis, and HCC (Grinberg et al., 2014). In our study, *ALDH8A1* was downregulated in HBx-overexpressing HepG2 cells and HBV-associated HCC patients. High expression of *ALDH8A1* is positively associated with OS in HBV-associated HCC patients, but its function in HBV-associated HCC is poorly understood.

Aldolase B (*ALDOB*), a crucial enzyme in glucose and fructose metabolism, is located on human chromosome 9q31.3 and encodes a protein of 40 kDa. Downregulated *ALDOB* is correlated with the absence of encapsulation, tumor size and early recurrence in HCC (Tao et al., 2015). In addition, low expression of *ALDOB* usually indicates a significantly shorter median of RFS and OS early-stage HCC patients. As a binding protein for the HBV S antigen (HBs), ALDOB-HBs interaction has been shown to inhibit cisplatin-induced apoptosis in HepG2 cells (Wu et al., 2014).

Angiopoietin like 6 (*ANGPTL6*) is located on human chromosome 19p13.2 and encodes a protein of 52 kDa. *ANGPTL6* has been identified as a circulating angiogenic factor that increases endothelial permeability and regulates angiogenesis (Oike et al., 2004; Santulli, 2014). Studies have found that high expression of *ANGPTL6* is a risk factor and associated with poor prognosis in HCC (Hu et al., 2021). However, in our study, *ANGPTL6* is downregulated in HBV-associated HCC, and high expression of *ANGPTL6* is positively correlated with OS in HBV-associated HCC.

Arginase 1 (*ARG1*) is located on human chromosome 6q23.2 and encodes a protein of 35 kDa. *ARG1* is a key factor in the urea cycle and transforms L-arginine to urea and L-ornithine, which is further metabolized to proline and polyamide metabolites that drive collagen synthesis and bioenergetic pathways in cell proliferation (Munder et al., 2005). The enzyme is specifically expressed in normal liver tissue. Therefore, it has specificity in the diagnosis of liver lesions (Lewin et al., 1987). Studies have shown lower levels of Arg-1 in patients with cirrhotic and HCC compared to that of healthy tissues (Chrzanowska, Krawczyk & Baranczyk-Kuzma, 2008). Arg-1 has high sensitivity and specificity in the diagnosis of HBV-associated HCC (Moudi, Mahmoudzadeh-Sagheb & Heidari, 2020). In our study, *ARG1* was downregulated in HBx-overexpressing HepG2 cells and HBV-associated HCC, but high expression level of *ARG1* was positively associated with the OS and RFS of patients with HBV-associated HCC. This finding suggests that *ARG1* is a good prognostic indicator in patients with HBV-associated HCC, but its function in HBV-associated HCC is poorly understood and needs further study.

Complement C8 alpha chain (*C8A*) is located on human chromosome 1p32.2 and encodes a protein of 65 kDa. *C8A* participates in the formation of the membrane attack complex (MAC) by forming pores in the plasma membrane of target cells, and MAC plays an important role in precursor and adaptive immune responses (Hadders, Beringer & Gros, 2007). It has been reported that *C8A* is identified as one potential biomarker for hepatocellular carcinogenesis form the HBV infection positive HCC datasets (Li et al., 2020b).

Cytoplasmic polyadenylation element binding protein 3 (*CPEB3*) is located on human chromosome 10q23.32 and encodes a protein of 76 kDa. *CPEB3* is a sequence-specific RNA-binding protein that acts as a translational repressor in the basal unstimulated state (Peng et al., 2010). It is downregulated in HCC tissue and is considered a good prognosis marker that effectively predicts OS (Yan et al., 2019; Zhang et al., 2020).

Family with sequence similarity 110 member C (*FAM110C*) is located on human chromosome 2p25.3 and encodes a protein of 34 kDa. FAM110C plays a role in cell spreading and migration of epithelial cells and has also been shown to be a marker of granulosa cell differentiation (Hauge, Patzke & Aasheim, 2007; Li et al., 2012). Recent studies have found that FAM110C is a unique gene that differentiates HBV infection from HBV-associated HCC and is expected to be a novel target for the development and prognosis of HBV-associated HCC (Wang et al., 2021). Similarly, in the results of our study, the high mRNA expression level of *FAM110C* was positively associated with the OS and RFS of patients with HBV-positive HCC.

G protein subunit alpha L (*GNAL*) is located on human chromosome 18p11.21 and encodes a protein of 44 kDa. *GNAL* is a stimulatory G protein alpha subunit that mediates odorant signaling in the olfactory epithelium. The novel mutated gene *GNAL* has the highest between coefficients in HCC samples (Zhang et al., 2014).The melanoma antigen gene (*MAGE*) family is a large, highly conserved group of proteins that have been reported to be involved in a variety of cancers in humans. *MAGEA6* is located on human chromosome Xq28 and encodes a protein of 35 kDa. It is upregulated and associated with worse prognosis in HCC patients (Li et al., 2020a).

Regulator of G protein signaling 5 (*RGS5*) is located on human chromosome 1q23.3 and encodes a protein of 21 kDa. *RGS5* is a member of the RGS protein family, and acts as a GTPase-activating protein of G protein *α* subunits, negatively regulating G-protein signal transduction (Hepler, 1999). Compared with normal controls, *RGS5* is high expressed in most HCC tissues or cell lines, and the expression level of *RGS5* is associated with poor OS in HCC patients (Hu et al., 2013). Immunohistochemical staining for RGS5 in 60 HCC patients shows that the protein level of RGS5 in cancer tissues of HCC patients is 63.3% higher than that in paired noncancerous tissue (Umeno et al., 2017). Similarly, the expression of *RGS5* is positively correlated with the degree of differentiation of gastric cancer (Wang et al., 2010).

Tyrosine aminotransferase (*TAT*) is located on human chromosome 16q22.2 and encodes a protein of 50 kDa. *TAT* is a mitochondrial protein in liver and catalyzes the conversion of L-tyrosine to p-hydroxyphenylpyruvate (Pasternack et al., 2009). It is downregulated and associated with poor prognosis in HCC patients (Nguyen, Nguyen & Le, 2020; Wang et al., 2019). In our study, *TAT* was downregulated in HBx-overexpressing HepG2 cells, HepG2.2.15 cells and HBV-associated HCC, and meanwhile low expression level of *TAT* was negatively associated with the OS and RFS of patients with HBV-positive HCC. These results suggests that *TAT* may be a good prognostic indicator in patients with HBV-positive HCC, but further studies are needed.

Even more mechanism research should be carried to further elucidate the relationship between these identified hub genes and hepatocellular carcinogenesis, we still believed that they were involved in the pathological processes of HBx-driven hepatocellular carcinogenesis although not all the candidate genes are the novel HCC associated genes.

## Conclusions

Overall, we identified that twelve candidate HBx-associated hub genes refer to the development and progression of HCC. In particular, *ARG1* and *TAT* are primarily expressed in liver and high expression of *ARG1* and *TAT* is positively related with OS and RFS in HBV-associated HCC patients. Additional, *ALDH8A1, ALDOB* and *FAM110C* are expected to be potential biomarkers of HCC, though further experimental verification is needed to confirm their potential prognostic value in HBV-positive HCC.

## Supplemental Information

10.7717/peerj.12697/supp-1 Supplemental Information 1 Results of GO and KEGG pathway enrichment analysis of HBx-associated DEGs (A) List the top ten items. The *x*-axis indicates -log10(P value). The right *y*-axis indicates the classification of GO. The left *y*-axis shows the functional annotations. (B) Bubble plot for KEGG enrichment results. The enrichment pathways are shown in the bubble diagram. GO, Gene Ontology. KEGG, Kyoto Encyclopedia of Genes and Genomes.

10.7717/peerj.12697/supp-2 Supplemental Information 2 Protein-protein interaction (PPI) network of HBx-associated DEGs (A) The PPI network of DEGs was constructed by Cytoscape. (B) The important module was based on PPI network with 15 nodes and 63 edges. Up-regulated genes are marked in red; down-regulated genes are marked in green.

10.7717/peerj.12697/supp-3 Supplemental Information 3 The mRNA expression of hub genes and HBx in SMMC-7721 cells of HBx transient overexpression. (A) *Aldh8a1*. (B) *Aldob*. (C) *Angptl6*. (D) *Arg1*. (E) *Cpeb3*. (F) *Fam110c*. (G) *Gnal*. (H) *Palm2*. (I) *Rgs5*. (J) *Tat*. (K) *HBx*.

10.7717/peerj.12697/supp-4 Supplemental Information 4 The mRNA expression of hub genes in the mouse model of HBx transient overexpression (A) *Aldh8a1*. (B) *Aldob*. (C) *Angptl6*. (D) *Arg1*. (E) *C8a*. (F) *Cpeb3*. (G) *Fam110c*. (H) *Gnal*. (I) *Palm2*. (J) *Rgs5*. (K) *Tat*.

10.7717/peerj.12697/supp-5 Supplemental Information 5 The mRNA expression of hub genes in primary mouse hepatocyte of HBx transient overexpression (A) *Aldh8a1*. (B) *Aldob*. (C) *Angptl6*. (D) *Arg1*. (E) *C8a*. (F) *Cpeb3*. (G) *Fam110c*. (H) *Gnal*. (I) *Palm2*. (J) *Rgs5*. (K) *Tat*. (L) *HBx*.

10.7717/peerj.12697/supp-6 Supplemental Information 6 Immunohistochemical staining from Human Protein Atlas and Oncogenic array analysis (A) Immunohistochemical staining for ALDH8A1, ALDOB, ARG1, FAM110C and GNAL of HCC and normal liver tissues. (B) Oncogenic array analysis in vector and HBx group revealed by Human XL Oncology Array Kit.

10.7717/peerj.12697/supp-7 Supplemental Information 7 Raw data for Fig. 1A

10.7717/peerj.12697/supp-8 Supplemental Information 8 Raw data for Fig. 1B

10.7717/peerj.12697/supp-9 Supplemental Information 9 Raw data for Figures 2B-2M-1

10.7717/peerj.12697/supp-10 Supplemental Information 10 Raw data for Figures 2B-2M-2

10.7717/peerj.12697/supp-11 Supplemental Information 11 Raw data for Figs. 3 and S3-1

10.7717/peerj.12697/supp-12 Supplemental Information 12 Raw data for Figs. 3, S3-2

10.7717/peerj.12697/supp-13 Supplemental Information 13 Raw data for Fig. S4-1

10.7717/peerj.12697/supp-14 Supplemental Information 14 Raw data for Fig. S4-2

10.7717/peerj.12697/supp-15 Supplemental Information 15 Raw data Figure for S4-3

10.7717/peerj.12697/supp-16 Supplemental Information 16 Raw data for Fig. S5

10.7717/peerj.12697/supp-17 Supplemental Information 17 Raw data for Fig. S6

10.7717/peerj.12697/supp-18 Supplemental Information 18 HepG2 cells STR identification

10.7717/peerj.12697/supp-19 Supplemental Information 19 Author_Checklist

## Additional Information and Declarations

### Data Availability

The following information was supplied regarding data availability: The raw data are available in the Supplementary Files.
