# Supplementary figures and images for: Transcriptomic identification of HBx-associated hub genes in hepatocellular carcinoma
Source: PeerJ. 2021 Dec 22;9:e12697. doi: 10.7717/peerj.12697 (PMC8710059; doi:10.7717/peerj.12697)

A

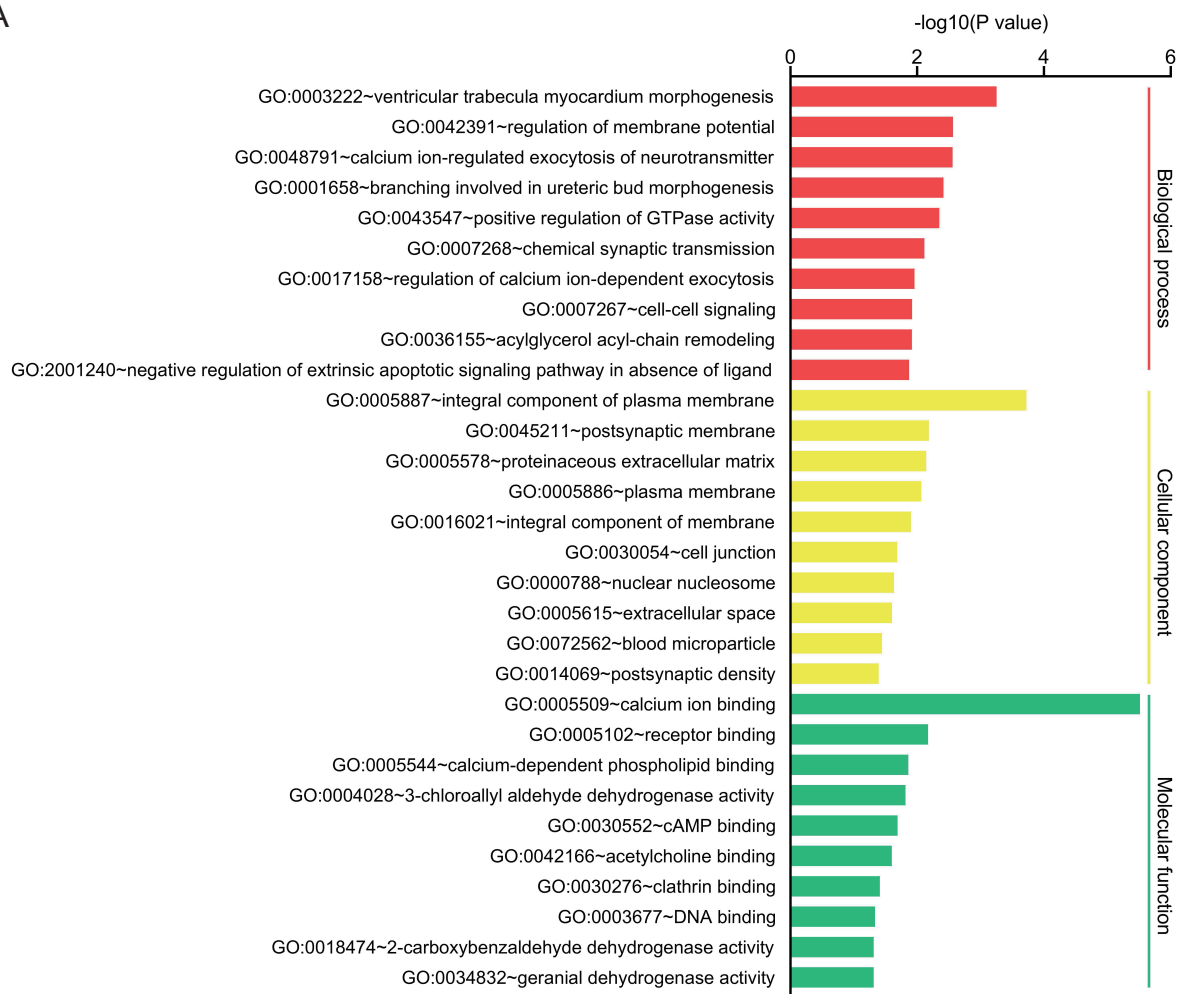

B

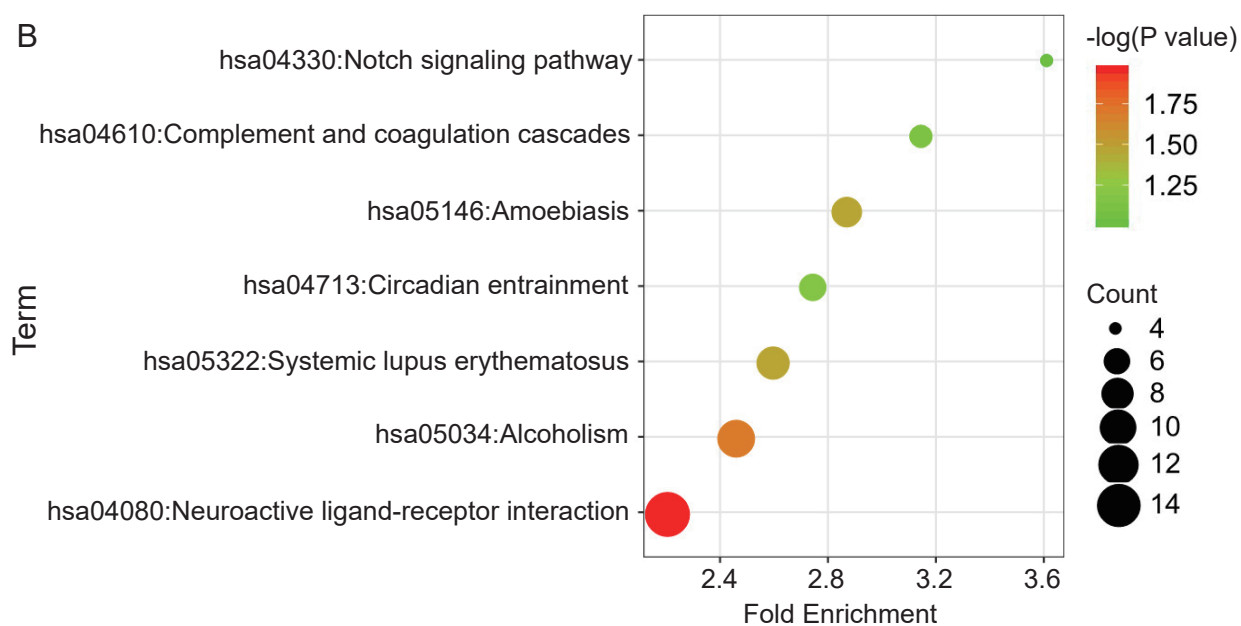

Supplement: Supplemental Information 1 — (A) List the top ten items. The x-axis indicates -log10(P value). The right y-axis indicates the classification of GO. The left y-axis shows the functional annotations. (B) Bubble plot for KEGG enrichment results. The enrichment pathways are shown in the bubble diagram. GO, Gene Ontology. KEGG, Kyoto Encyclopedia of Genes and Genomes. [file peerj-09-12697-s001.pdf]

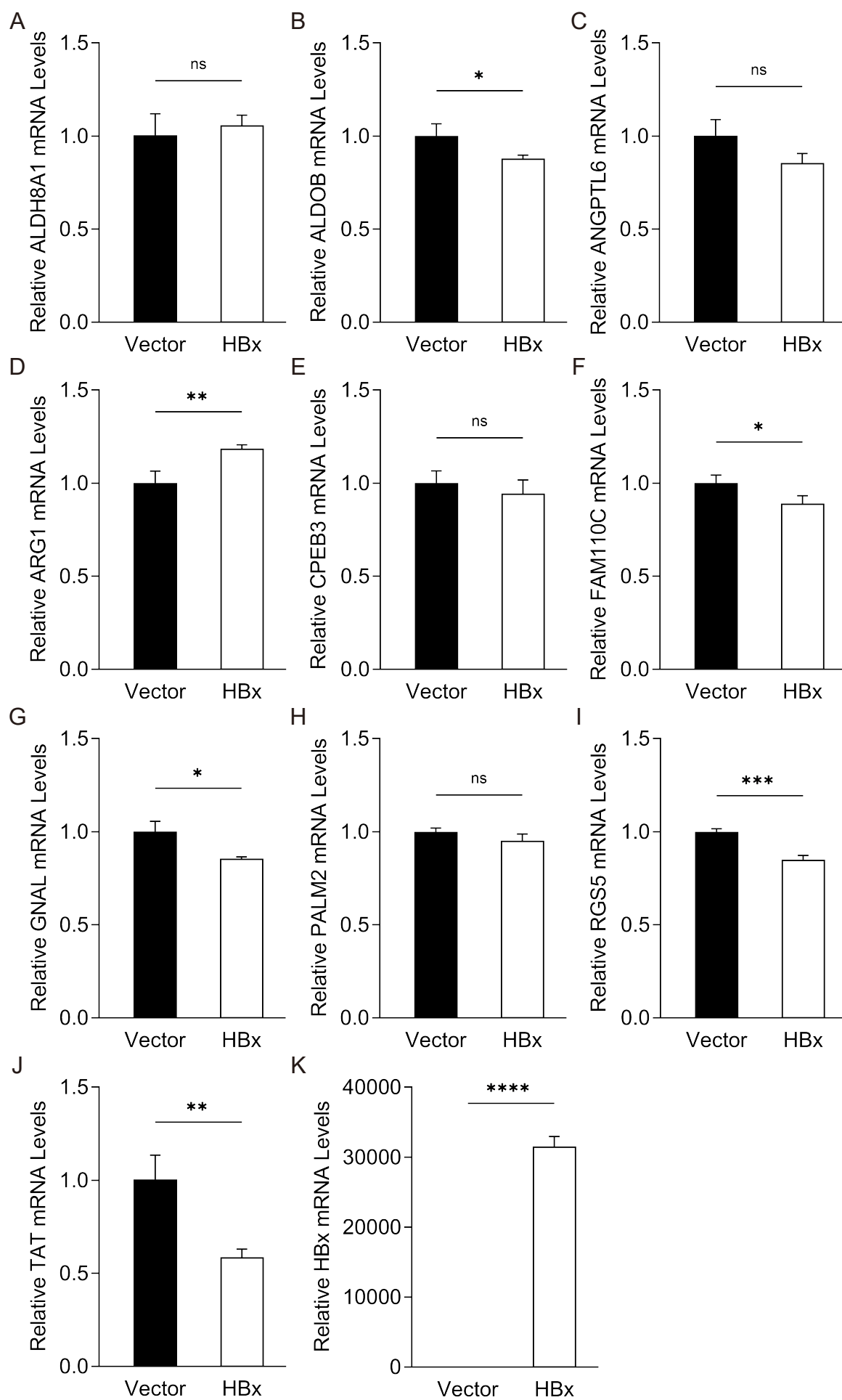

Supplement: Supplemental Information 3 — (A) Aldh8a1. (B) Aldob. (C) Angptl6. (D) Arg1. (E) Cpeb3. (F) Fam110c. (G) Gnal. (H) Palm2. (I) Rgs5. (J) Tat. (K) HBx. [file peerj-09-12697-s003.pdf]

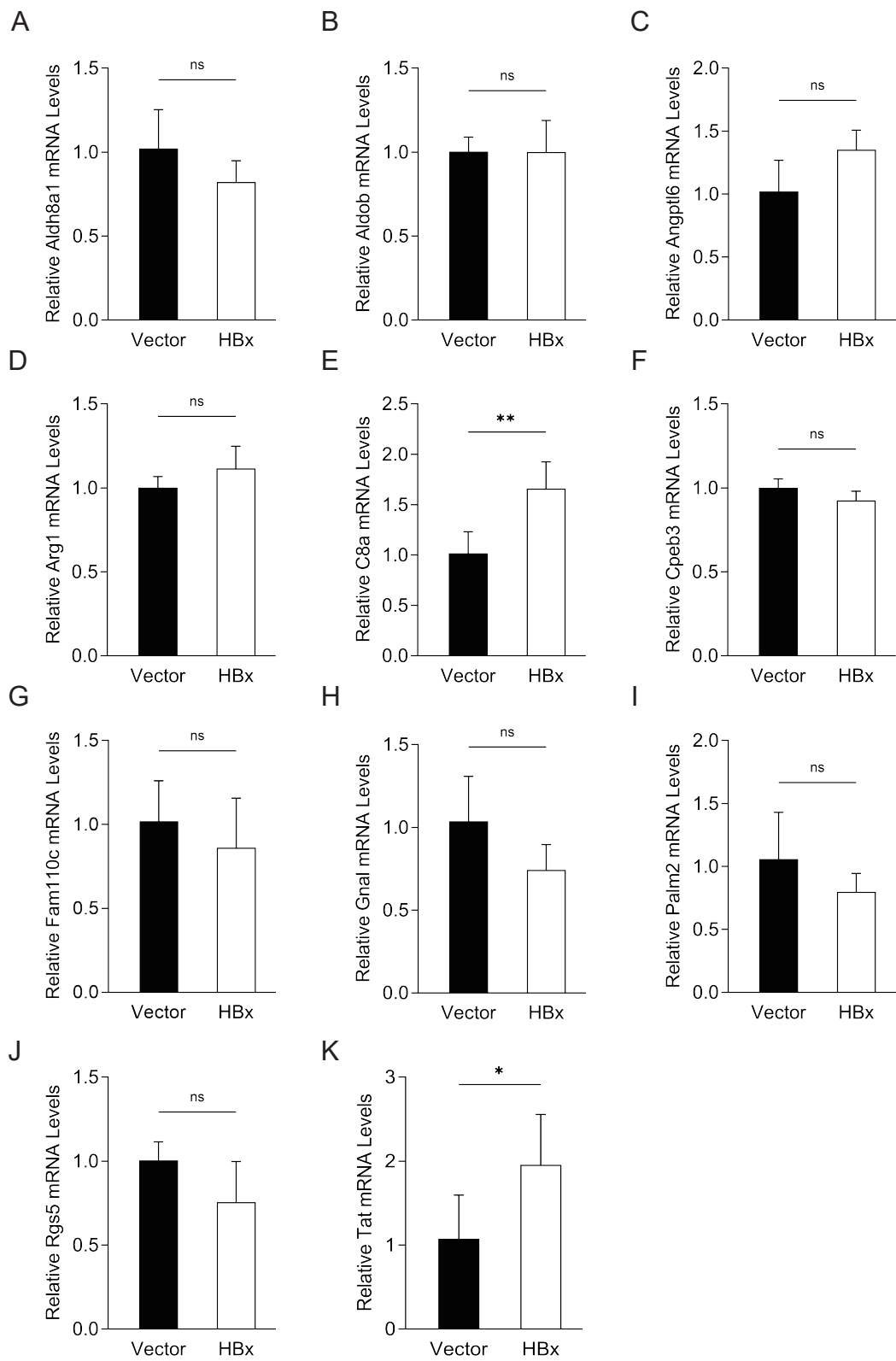

Supplement: Supplemental Information 4 — (A) Aldh8a1. (B) Aldob. (C) Angptl6. (D) Arg1. (E) C8a. (F) Cpeb3. (G) Fam110c. (H) Gnal. (I) Palm2. (J) Rgs5. (K) Tat. [file peerj-09-12697-s004.pdf]

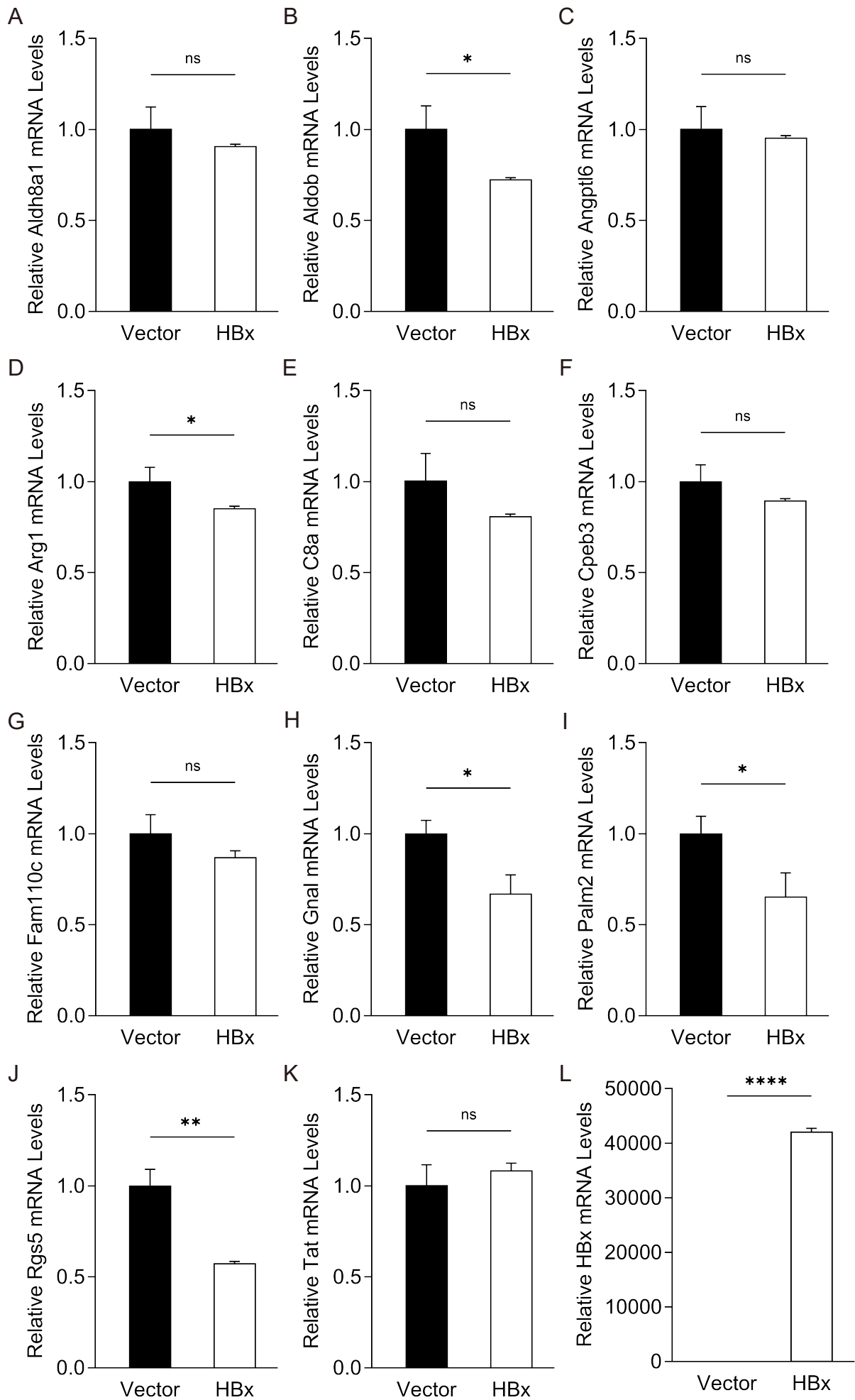

Supplement: Supplemental Information 5 — (A) Aldh8a1. (B) Aldob. (C) Angptl6. (D) Arg1. (E) C8a. (F) Cpeb3. (G) Fam110c. (H) Gnal. (I) Palm2. (J) Rgs5. (K) Tat. (L) HBx. [file peerj-09-12697-s005.pdf]

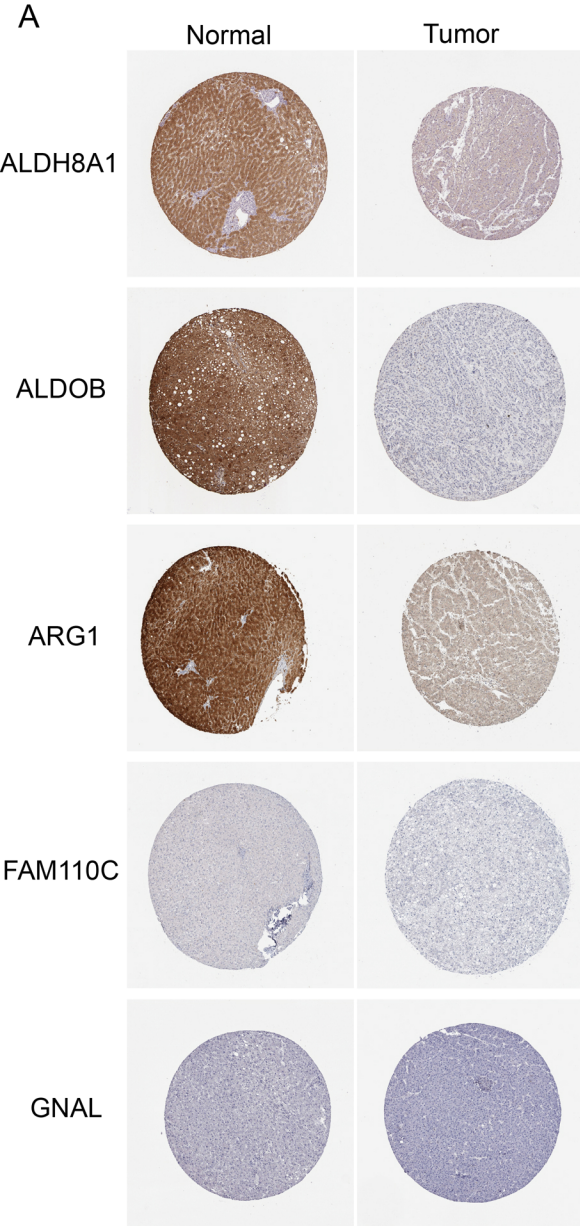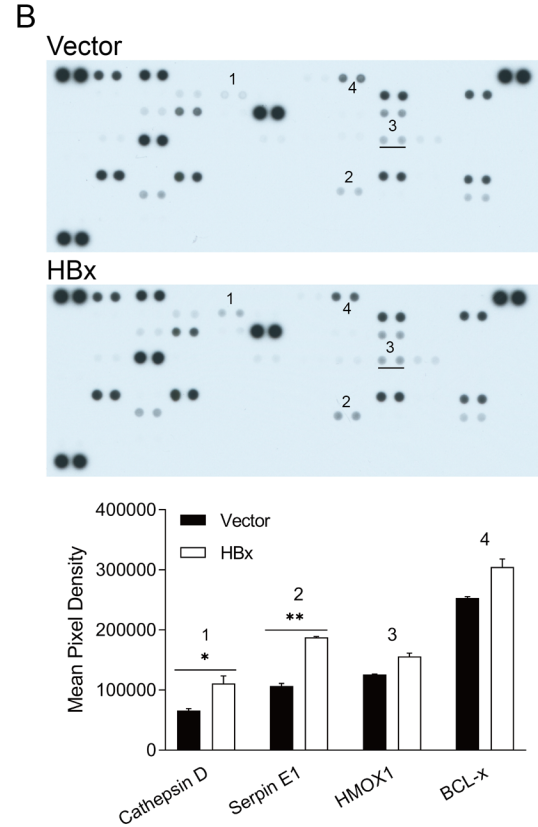

Supplement: Supplemental Information 6 — (A) Immunohistochemical staining for ALDH8A1, ALDOB, ARG1, FAM110C and GNAL of HCC and normal liver tissues. (B) Oncogenic array analysis in vector and HBx group revealed by Human XL Oncology Array Kit. [file peerj-09-12697-s006.pdf]

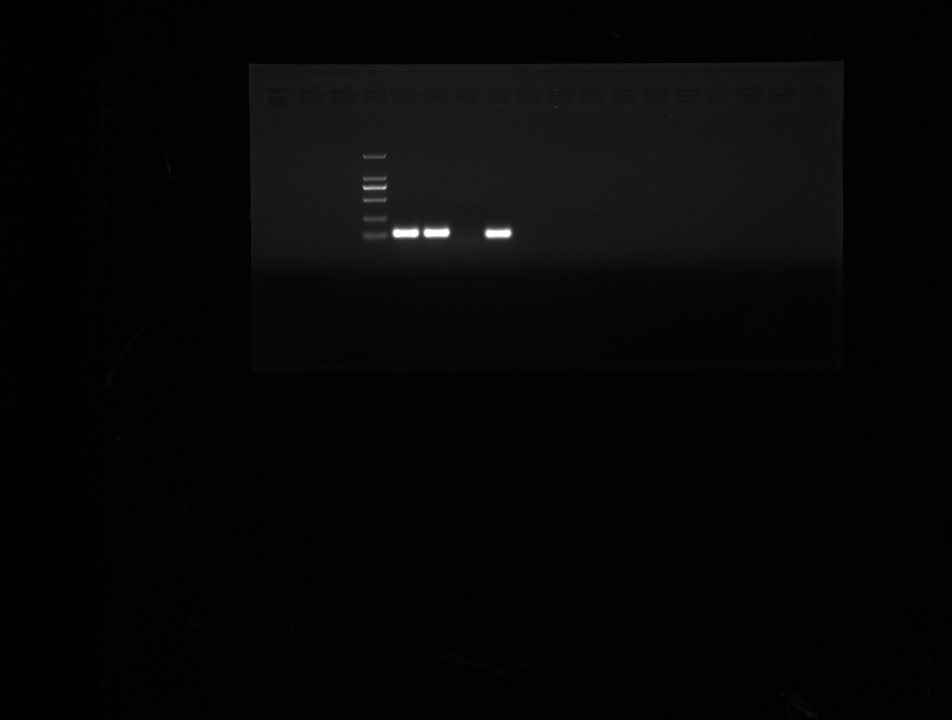

Supplement: Supplemental Information 7 [file peerj-09-12697-s007.jpg]

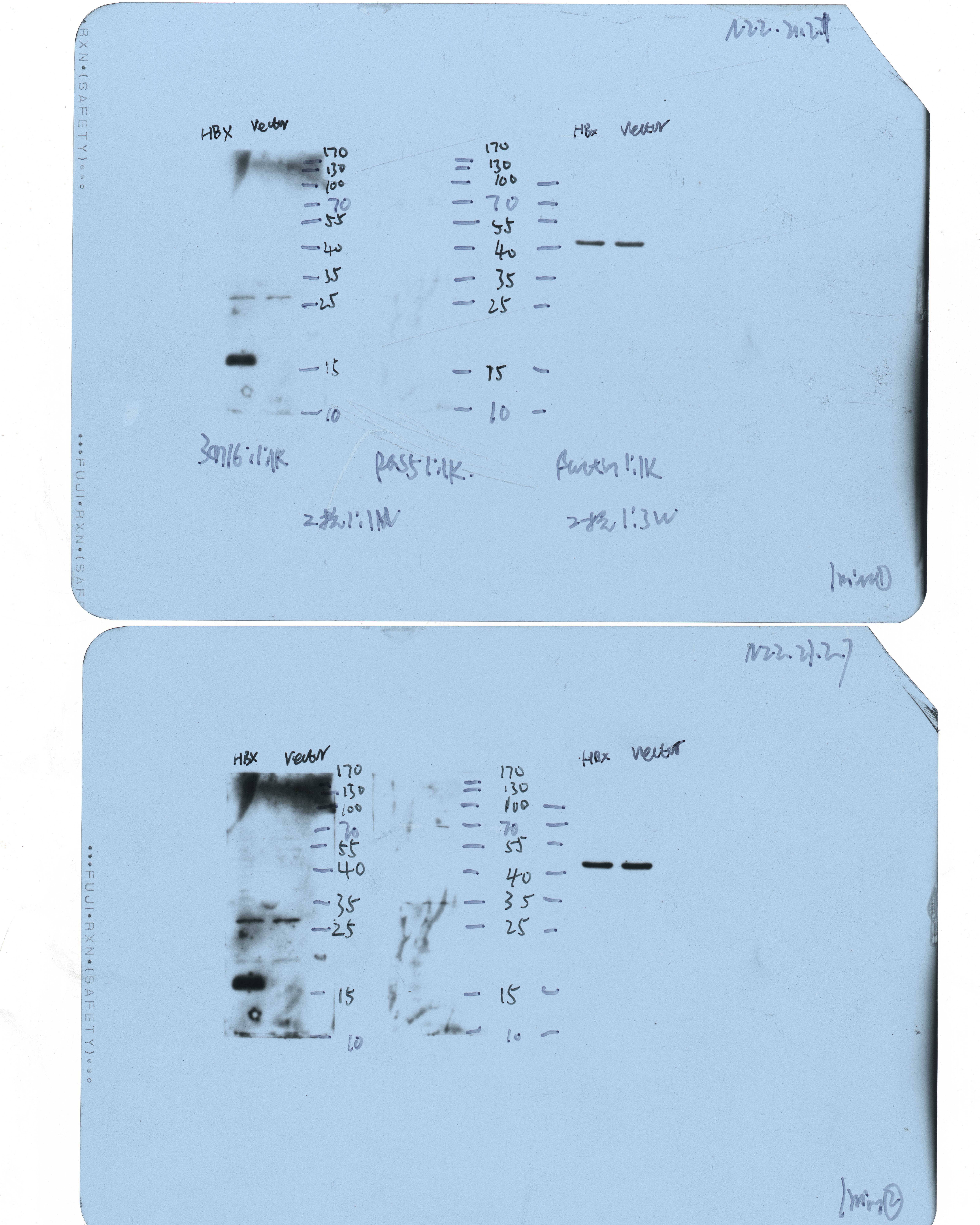

Supplement: Supplemental Information 8 [file peerj-09-12697-s008.jpg]

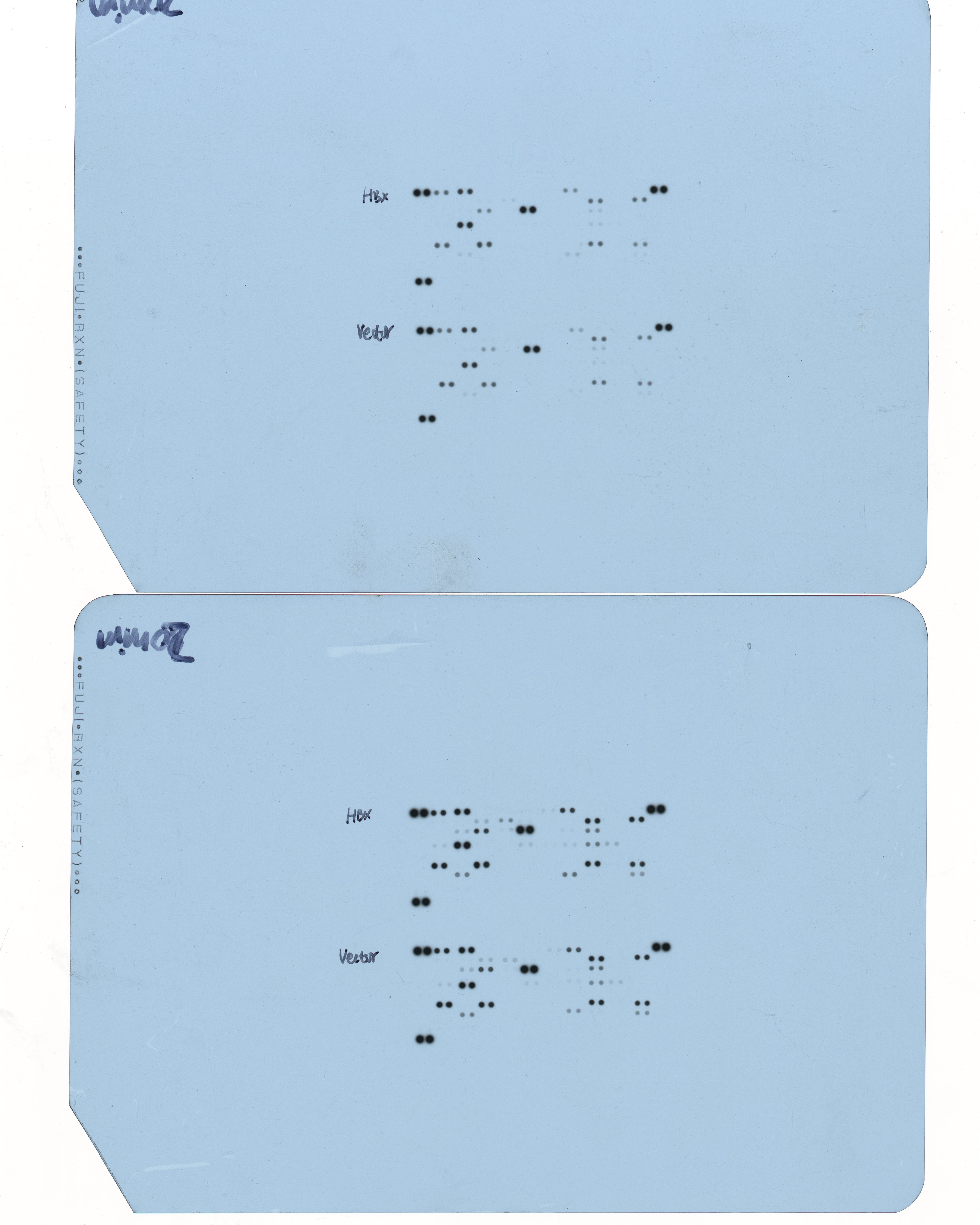

Supplement: Supplemental Information 17 [file peerj-09-12697-s017.jpg]
