# Supplementary material for: Transcriptomic identification of HBx-associated hub genes in hepatocellular carcinoma
Source: PeerJ. 2021 Dec 22;9:e12697. doi: 10.7717/peerj.12697 (PMC8710059; doi:10.7717/peerj.12697)
Supplement: Supplemental Information 2 — (A) The PPI network of DEGs was constructed by Cytoscape. (B) The important module was based on PPI network with 15 nodes and 63 edges. Up-regulated genes are marked in red; down-regulated genes are marked in green. [file peerj-09-12697-s002.pdf]

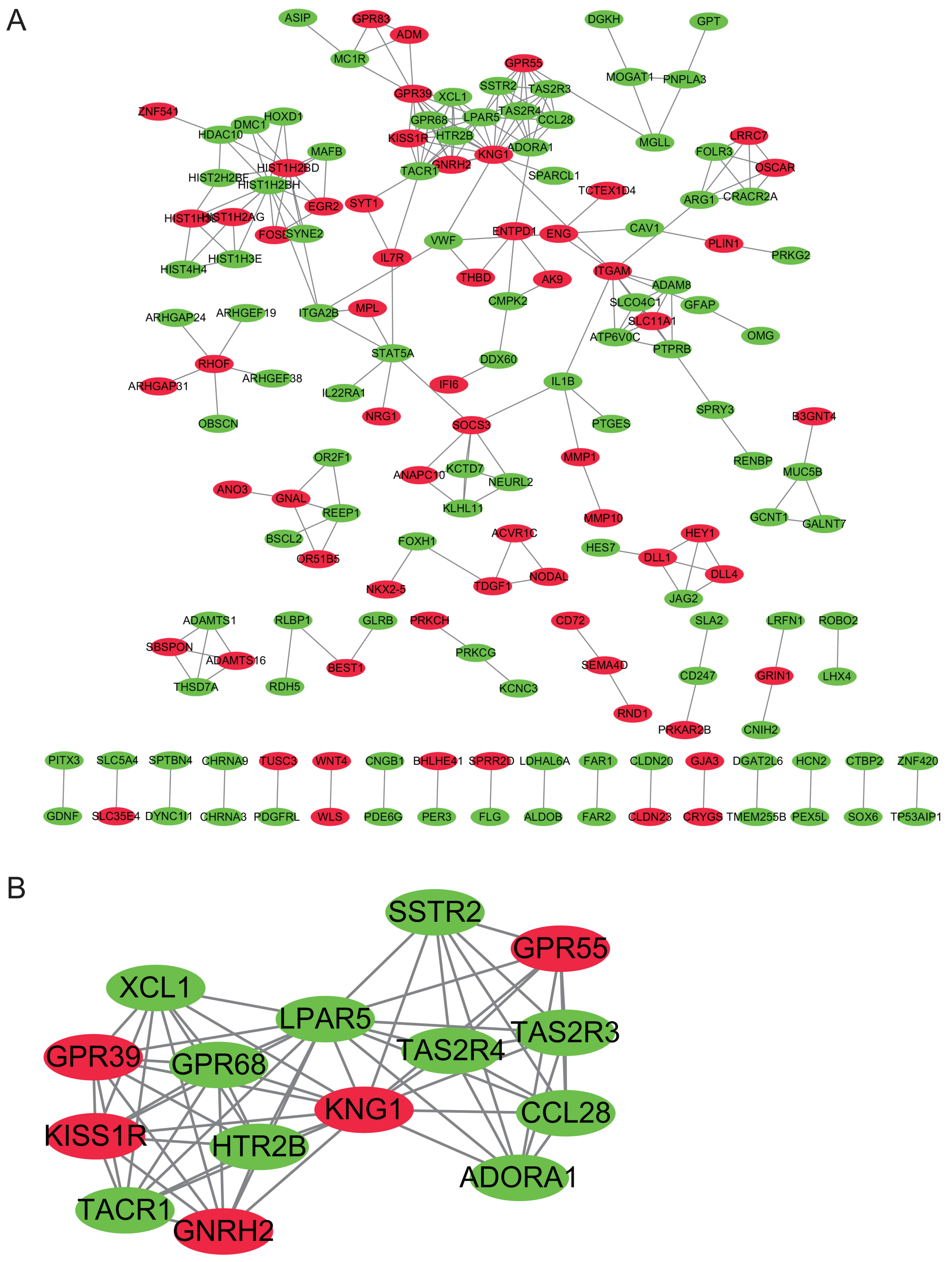

A
ASIP
GPR83
ADM
MC1R
DGKH
GPT
GPR55
MOGAT1
PNPLA3
SSTR2
TAS2R3
GPR39
XCL1
TAS2R4
ZNF541
HOXD1
DMC1
GPR68
LPAR5
CCL28
HDAC10
KISS1R
HTR2B
ADORA1
MGLL
LRRC7
MAFB
KNG1
FOLR3
HIST1H2BD
TACR1
GNRH2
OSCAR
HIST2H2BF
HIST1H2BH
SPARCL1
TCTEX1D4
CRACR2A
ARG1
EGR2
SYT1
HIST1H2AG
SYNE2
ENTPD1
CAV1
VWF
ENG
PLIN1
HIST1H3E
IL7R
PRKG2
HIST4H4
THBD
AK9
ITGAM
ADAM8
CMPK2
SLCO4C1
GFAP
ARHGAP24
ARHGEF19
ITGA2B
MPL
SLC11A1
ATP6V0C
OMG
PTPRB
STAT5A
DDX60
RHOF
ARHGEF38
ARHGAP31
IFI6
IL1B
IL22RA1
OBSCN
NRG1
SOCS3
PTGES
SPRY3
B3GNT4
RENBP
OR2F1
MMP1
MUC5B
ANAPC10
KCTD7
ANO3
GNAL
NEURL2
KLHL11
REEP1
BSCL2
MMP10
GCNT1
GALNT7
OR51B5
FOXH1
ACVR1C
HEY1
HES7
DLL1
DLL4
NKX2-5
TDGF1
NODAL
JAG2
ADAMTS1
RLBP1
GLRB
PRKCH
CD72
SLA2
LRFN1
ROBO2
SBSPON
ADAMTS16
PRKCG
BEST1
SEMA4D
CD247
GRIN1
LHX4
THSD7A
RDH5
KCNC3
RND1
PRKAR2B
CNIH2
PITX3
SLC5A4
SPTBN4
CHRNA9
TUSC3
WNT4
CNGB1
BHLHE41
SPRR2D
LDHAL6A
FAR1
CLDN20
GJA3
DGAT2L6
HCN2
CTBP2
ZNF420
GDNF
SLC35E4
DYNC1I1
CHRNA3
PDGFRL
WLS
PDE6G
PER3
FLG
ALDOB
FAR2
CLDN23
CRYGS
TMEM255B
PEX5L
SOX6
TP53AIP1
B
SSTR2
GPR55
XCL1
LPAR5
TAS2R3
GPR39
GPR68
TAS2R4
KNG1
CCL28
KISS1R
HTR2B
ADORA1
TACR1
GNRH2
